# Nitric oxide activates AMPK by modulating PDE3A in human pulmonary artery smooth muscle cells

**DOI:** 10.14814/phy2.14559

**Published:** 2020-09-10

**Authors:** Julie Dillard, Xiaomei Meng, Leif Nelin, Yusen Liu, Bernadette Chen

**Affiliations:** ^1^ Pulmonary Hypertension Group Center for Perinatal Research Abigail Wexner Research Institute at Nationwide Children's Hospital Columbus OH USA; ^2^ Department of Pediatrics The Ohio State University College of Medicine Columbus OH USA

**Keywords:** cyclic nucleotides, persistent pulmonary hypertension of the newborn, phosphodiesterase, pulmonary hypertension, soluble guanylate cyclase

## Abstract

Phosphodiesterase 3 (PDE3), of which there are two isoforms, PDE3A and PDE3B, hydrolyzes cAMP and cGMP—cyclic nucleotides important in the regulation of pulmonary vascular tone. PDE3 has been implicated in pulmonary hypertension unresponsive to nitric oxide (NO); however, contributions of the two isoforms are not known. Furthermore, adenosine monophosphate‐activated protein kinase (AMPK), a critical regulator of cellular energy homeostasis, has been shown to be modulated by PDE3 in varying cell types. While AMPK has recently been implicated in pulmonary hypertension pathogenesis, its role and regulation in the pulmonary vasculature remain to be elucidated. Therefore, we utilized human pulmonary artery smooth muscle cells (hPASMC) to test the hypothesis that NO increases PDE3 expression in an isoform‐specific manner, thereby activating AMPK and inhibiting hPASMC proliferation. We found that in hPASMC, NO treatment increased PDE3A protein expression and PDE3 activity with a concomitant decrease in cAMP concentrations and increase in AMPK phosphorylation. Knockdown of PDE3A using siRNA transfection blunted the NO‐induced AMPK activation, indicating that PDE3A plays an important role in AMPK regulation in hPASMC. Treatment with a soluble guanylate cyclase (sGC) stimulator increased PDE3A expression and AMPK activation similar to that seen with NO treatment, whereas treatment with a sGC inhibitor blunted the NO‐induced increase in PDE3A and AMPK activation. These results suggest that NO increases PDE3A expression, decreases cAMP, and activates AMPK via the sGC‐cGMP pathway. We speculate that NO‐induced increases in PDE3A and AMPK may have implications in the pathogenesis and the response to therapies in pulmonary hypertensive disorders.

## Graphical abstract

Nitric oxide treatment increased PDE3A protein expression and PDE3 activity with a concomitant decrease in cAMP concentrations and increase in AMPK phosphorylation in human pulmonary artery smooth muscle cells. Knockdown of PDE3A using siRNA transfection blunted the NO‐induced AMPK activation, indicating that PDE3A plays an important role in AMPK regulation in hPASMC. We speculate that NO‐induced increases in PDE3A and AMPK may have implications in the pathogenesis of pulmonary hypertensive disorders.

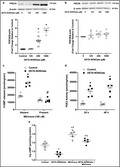

## 1 INTRODUCTION

Pulmonary hypertension is a complex, multifactorial disease characterized by vascular remodeling and inflammation. In neonates, persistent pulmonary hypertension of the newborn (PPHN) results when the normal circulatory transition at birth fails to occur. In PPHN, the pulmonary pressures remain elevated causing right‐to‐left shunting across the ductus arteriosus and foramen ovale. This shunting of blood results in hypoxemic respiratory failure, which can lead to eventual right heart failure and death (Lakshminrusimha, Mathew, & Leach, 2016; Nair & Lakshminrusimha, 2014). Used in combination with supplemental oxygen, inhaled nitric oxide (NO) is the only Food and Drug Administration‐approved therapy for PPHN. However, up to 40% of patients do not respond to these interventions, and mortality is high, particularly for the subgroup of NO nonresponders (Clark et al., 2000; Finer & Barrington, 2006; Group NINOS, 1997). Hence, there is a critical need to elucidate the mechanistic pathways that underlie this disease. Although the use of the phosphodiesterase 3 (PDE3) inhibitor, milrinone, to treat PPHN is increasing, an understanding of the interactions between the PDE3‐cyclic adenosine 3′5′‐monophosphate (cAMP) and the NO‐cyclic guanosine 3′,5′‐monophosphate (cGMP) pathways in PPHN is limited.

The second messengers, cAMP and cGMP, regulate vascular tone in the lungs. They are synthesized by adenylate and guanylate cyclases, respectively, while the cyclic nucleotide PDEs degrade both cAMP and cGMP. NO activates the enzyme soluble guanylate cyclase (sGC), which converts guanosine 5'‐triphosphate (GTP) to cGMP. This results in decreased intracellular calcium stores, thereby promoting vasodilation (Rose, Liu, Palmer, & Maurice, 1997; Rybalkin, Yan, Bornfeldt, & Beavo, 2003). The PDE3 family of PDEs, of which there are two isoforms, PDE3A and PDE3B, hydrolyzes both cGMP and cAMP; however, they exhibit a higher affinity and lower rate of catalytic hydrolysis for cGMP than for cAMP. Thus, cGMP acts as a competitive inhibitor of cAMP hydrolysis by PDE3 (Degerman, Belfrage, & Manganiello, 1997; Manganiello, Taira, Degerman, & Belfrage, 1995). Consequently, an increase in cGMP levels by NO would be expected to inhibit PDE3 hydrolysis of cAMP. Despite this, animal models show increased PDE3 expression/activity following treatment with NO and enhanced vasorelaxation of the pulmonary vasculature with PDE3 inhibition (Busch et al., 2010; Chen, Lakshminrusimha, et al., 2009; Lakshminrusimha et al., 2009; Murray, MacLean, & Pyne, 2002; Thelitz et al., 2004). Additional animal studies have implicated PDE3 as the reason for the observed rebound pulmonary hypertension when NO is discontinued (Byers et al., 2012; Namachivayam et al., 2006; Thelitz et al., 2004).

PDE3A and PDE3B exhibit differences in biochemical properties, molecular regulation, biological function, and cell type specificities (Degerman et al., 1997). PDE3 isoforms have been shown to regulate adenosine monophosphate‐activated protein kinase (AMPK), a critical regulator of cellular energy homeostasis, in varying cell types (Omar, Zmuda‐Trzebiatowska, Manganiello, Göransson, & Degerman, 2009; Suzuki, Uchida, Nakanishi, & Hattori, 2008). Moreover, AMPK has recently been implicated in pulmonary hypertension pathogenesis. However, the distinct roles of each PDE3 isoform and their regulation of AMPK in pulmonary hypertensive diseases remain to be elucidated, in part, because the only existing PDE3 inhibitors are not isoform‐specific.

In this study, we utilized human pulmonary artery smooth muscle cells (hPASMC) to test the hypothesis that NO increases PDE3 expression and/or activity, which, in turn, leads to AMPK phosphorylation/activation. Importantly, we determined the distinct role of each PDE3 isoform on NO‐induced AMPK phosphorylation in hPASMC. Finally, in light of the controversies in the literature on the role of AMPK in pulmonary hypertension, we studied the contribution of AMPK in vascular smooth muscle cell proliferation in our cell culture model.

## 2 METHODS

### 2.1 Chemicals

Unless otherwise indicated, all chemicals were obtained from Sigma‐Aldrich (St. Louis, MO). BAY 41‐2272 (3‐(4‐Amino‐5‐cyclopropylpyrimidin‐2‐yl)‐1‐(2‐fluorobenzyl)‐1H‐pyrazolo[3,4‐b]pyridine) is a sGC agonist; ODQ (1H‐[1,2,4]Oxadiazolo[4,3‐a]quinoxalin‐1‐one) is a selective inhibitor of NO‐sensitive sGC; and milrinone (1,6‐Dihydro‐2‐methyl‐6‐oxo‐(3,4′‐bipyridine)‐5‐carbonitrile) is a PDE3 inhibitor. DETA NONOate ((Z)‐1‐[N‐(2‐aminoethyl)‐N‐(2‐ammonioethyl)amino]diazen‐1‐ium‐1,2‐diolate), an NO donor, was purchased from Cayman Chemical (Ann Arbor, MI). AICAR (5‐Aminoimidazole‐4‐carboxamide‐1‐β‐D‐ribofuranoside), an AMPK agonist, was purchased from Santa Cruz Biotechnology (Santa Cruz, CA).

### 2.2 Cell culture and cell lysis

Human PASMC (ScienCell, catalog #3110, lot #7449, 4551; Carlsbad, CA) were cultured in complete smooth muscle cell media (ScienCell) in 5% CO_2_ at 37°C as previously described (Chen, Calvert, Cui, & Nelin, 2009a). Human PASMC between passages 6–9 were used in experiments, and no significant changes in morphology were observed with these passages. On the day of the experiment, smooth muscle cells were fed with fresh culture medium after washing the hPASMC with Dulbecco's phosphate‐buffered saline (DPBS). Where indicated, the cells were treated with 125‐, 250‐, or 1,000‐μM DETA NONOate; 10‐µM BAY 41‐2272; 10‐µM ODQ; 10‐ or 100‐µM milrinone; or 5‐µM AICAR. DMSO or 0.1 M NaOH served as vehicle controls. In some studies, hPASMC were treated with an additional dose of milrinone (10 μM) at the 24‐hr time point. The cells were then incubated in 21% O_2_, 5% CO_2_, and balance N_2_ (normoxia) at 37°C.

After incubation for 24–48 hr, hPASMC were washed with DPBS and lysed in 50‐μl lysis buffer (Chen, Calvert, et al., 2009). The lysis buffer was comprised of 20‐mM HEPES, pH 7.4; 50‐mM β‐glycerophosphate; 2‐mM EGTA; 1‐mM DTT; 10‐mM NaF; 1‐mM Na_3_VO_4_; 1% Triton X‐100; and 10% glycerol. Thirty minutes before use, 1 μg/ml aprotinin, 1 μg/ml leupeptin, and 1‐mM phenylmethylsulfonyl fluoride were added to the lysis buffer to inhibit protease function. The hPASMC were scraped to aid lysis and placed on ice for 30 min. The cell lysates were then centrifuged (14,000 *g*, 15 min, 4°C). The supernatant was removed and stored at −80°C until use.

### 2.3 Protein isolation and Western blot

Protein concentration in the soluble lysates was determined by the Bradford method using a commercially available assay (Bio‐Rad, Hercules, CA) as previously described (Chen, Calvert, et al., 2009). PDE3A, PDE3B, phosphorylated (p) and total (T) AMPK, phosphorylated and total acetyl‐CoA carboxylase (ACC), and β‐actin in cell lysates were analyzed by Western blotting using mouse anti‐PDE3A (1:500; catalog #sc‐293446, lot #J0217), mouse anti‐PDE3B (1:250; catalog #sc‐376823, lot #J2516), rabbit anti‐p‐AMPK (1:500; catalog #sc‐33524, lot #C0813), rabbit anti‐T‐AMPK (1:500; catalog #sc‐25792, lot #A3013), rabbit anti‐p‐ACC (1:1,000; catalog #11818, lot #D7D11), or rabbit anti‐T‐ACC (1:1,000; catalog #3676, lot #C83B10) primary antibodies. Antibodies to PDE3A, PDE3B, p‐AMPK, and T‐AMPK were obtained from Santa Cruz Biotechnology; and antibodies to p‐ACC and T‐ACC were obtained from Cell Signaling Technology (Danvers, MA).

### 2.4 PDE3 activity

PDE3 activity was measured using a commercially available colorimetric PDE assay kit (Abcam, Cambridge, UK) as previously described (Chen, Lakshminrusimha, et al., 2009). Human PASMC were treated with DETA NONOate (250 µM) and incubated in normoxia for 24 or 48 hr. Total protein was prepared as described above. Free phosphate contamination was removed by using Centri‐Spin 10 columns (Princeton Separations, Adelphia, NJ). Protein was quantified as described above and 5‐µg protein was used for each sample. The reactions were started in a timed fashion. Each sample was read in duplicate in the presence or absence of milrinone (100 μM) to determine PDE3‐specific cAMP hydrolysis. Samples were incubated for 30 min at 30°C and stopped in a timed fashion by the addition of 100‐µl Green Assay Reagent. Samples were incubated on an orbital shaker at room temperature for 30 min. Results were measured using a Spectramax M2 automated microplate reader (Molecular Devices, LLC, San Jose, CA). The difference between the pmol 5′AMP measured with and without milrinone represents the PDE3‐specific cAMP hydrolytic activity.

### 2.5 cAMP ELISA

cAMP was measured in hPASMC lysates using a competitive, colorimetric ELISA kit (Abcam) as previously described (Chen, Lakshminrusimha, et al., 2009), according to the manufacturer's instructions. Human PASMC were incubated (48 hr, 37°C, 5% CO_2_) with or without DETA NONOate (250 µM) and/or milrinone (10 μM) before initiation of the assay. Untreated hPASMC served as controls. Samples were read using a Spectramax M2 microplate reader (Molecular Devices), and cAMP levels in treated and untreated cell lysates were determined by plotting the data obtained against a cAMP standard curve. Results are shown as picomole cAMP per milligram protein.

### 2.6 Transfection of siRNA against PDE3A and PDE3B

Small interfering (si) RNA transfection was used to determine the effects of PDE3A and PDE3B gene silencing on AMPK activation. Transient transfection of siRNA against PDE3A (siPDE3A) or PDE3B (siPDE3B) was performed with DharmaFECT 1 transfection reagent (Thermo Fisher Scientific, Lafayette, CO) according to the manufacturer's protocol. Briefly, in 1.5‐ml centrifuge tubes, 100 μl of 2‐μM siPDE3A (Dharmacon, catalog #M‐007646‐01‐0010), siPDE3B (Dharmacon, catalog #M‐007646‐01‐0050), or scramble siRNA (Santa Cruz, catalog #sc‐37007) was mixed with 100 μl of smooth muscle basal medium (ScienCell), vortexed, and incubated at room temperature for 5 min. The DharmaFECT 1 transfection reagent was diluted 1:100 (total volume of 200 μl) and incubated at room temperature for 5 min. The transfection reagent was then added to each sample of siRNA, mixed, and incubated at room temperature for 20 min. Smooth muscle growth media (ScienCell, 600 μl) were then added to each tube for a total volume of 1 ml (final siRNA concentration of 100 nM).

Human PASMC, grown to ~70% confluence in six‐well plates, were washed with DPBS. Fresh media (1 ml) + siRNA‐DharmaFECT reagent mixture (1 ml) was placed in each well and incubated for 48 hr. The media were then removed and cells were washed. DETA NONOate (250 μM) was added in 1.5‐ml fresh media to feed the hPASMC, and the hPASMC were incubated for an additional 24 hr.

### 2.7 MTT proliferation assay

Human PASMC proliferation was measured using dimethylthiazol (MTT). The assay was performed according to the manufacturer's (Sigma‐Aldrich) protocol. Briefly, DETA NONOate (250 µM), AICAR (5 µM), or milrinone (10 µM) was added to hPASMC cultures and incubated in normoxia at 37°C for 48 hr. MTT (0.5 mg/ml) was then added, and hPASMC were incubated for an additional 4 hr. The mitochondrial reductase in living cells reduces MTT to purple formazan, which is detected by spectrophotometry. Samples were read at 570 nm using a Spectramax M2 microplate reader (Molecular Devices). The measured absorbance directly correlates with the number of viable cells. The values obtained for DETA NONOate, AICAR, or milrinone‐treated cells were normalized to those values obtained for vehicle‐treated cells.

### 2.8 Statistical analysis

All of the studies were performed at least two separate times in duplicate or triplicate. In the figures, *n* represents the total number of data points for a given experiment. Values are presented as means ± *SEM*. Unpaired Student's *t* test and one‐way or two‐way ANOVA with post hoc Tukey's analyses for multiple comparisons were used to compare groups as appropriate (GraphPad Prism Software, La Jolla, CA). Differences were considered significant when *p* < .05.

## 3 RESULTS

### 3.1 DETA NONOate increased PDE3 expression and activity, with a concomitant decrease in cAMP concentrations

**Figure 1:**
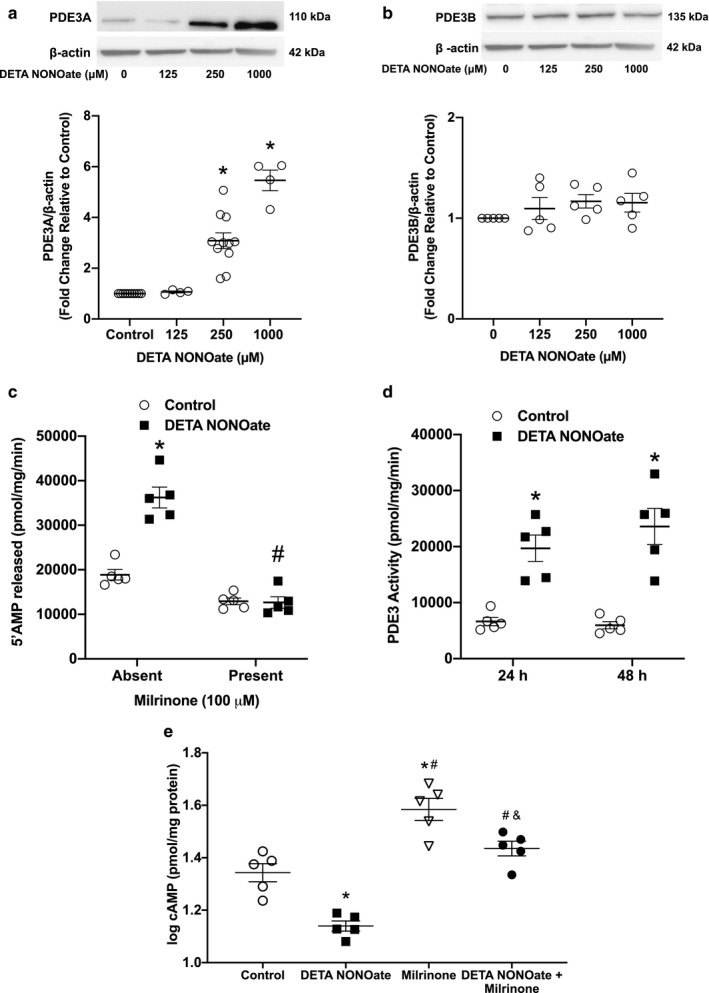
NO increased PDE3A protein expression and PDE3 activity with a concomitant decrease in cAMP concentrations in hPASMC. Human PASMC were treated with increasing concentrations of the NO donor, DETA NONOate, for 48 hr. Protein was harvested, analyzed by Western blot, and expression quantified by densitometry, assayed for PDE3 activity, or cAMP concentrations measured by ELISA. Representative Western blots with data shown below as fold changes ± *SEM* relative to vehicle control for (a) PDE3A and β‐actin; *n* = 4–11 for each group, *p < .001 different than control (one‐way ANOVA) and (b) PDE3B and β‐actin; *n* = 5 for each group. (c) 5′AMP‐released after hPASMC were treated with DETA NONOate (250 μM) for 48 hr in the absence or presence of milrinone (100 μM), reported as pmol 5′AMP/mg protein/min, * *p* < .001 different than control, absent milrinone; #*p* < .04 different from control, absent milrinone (two‐way ANOVA). (d) PDE3‐specific activity after hPASMC were treated with DETA NONOate (250 μM) for 24 or 48 hr, reported as the fraction of PDE activity inhibited by milrinone (pmol 5′AMP/mg protein/min); samples were analyzed in duplicate ± milrinone (100 μM) and shown as means ± *SEM*; *n* = 5 for each group, *p ≤ .002 different than control (two‐way ANOVA) and (e) cAMP concentrations after treatment with DETA NONOate (250 μM) for 48 hr in the absence or presence of milrinone (10 μM), log transformed reported as pmol/mg protein; *n* = 5 for each group, *p < .002 different from control, #*p* < .001 different from DETA NONOate, &*p* < .03 different from milrinone (log transformed, one‐way ANOVA)

Human PASMC were treated with the NO donor, DETA NONOate, to define the effect of NO on the expression of PDE3 isoforms as well as on PDE3 activity at a confluency of ~ 80%. We quantified PDE3A and PDE3B protein levels in hPASMC lysates by Western blot and densitometry. We found that treatment with DETA NONOate for 48 hr increased PDE3A protein expression in a dose‐dependent manner relative to the vehicle control (Figure 1a). An approximate three‐ and fivefold increase in PDE3A protein expression was observed with the addition of 250‐μM and 1‐mM DETA NONOate, respectively (*p* < .001, Figure 1a). There was no significant change in the expression of PDE3B (Figure 1b).

Given that protein expression may not be synonymous with protein function, we measured PDE3 enzymatic activity using a commercially available assay kit. Human PASMC were treated with DETA NONOate (250 µM) or control. The milrinone‐inhibitable fraction of total cAMP PDE activity represents PDE3 enzyme activity. The 5′AMP released from hPASMC was found to be significantly greater after 48‐hr treatment with DETA NONOate (Figure 1c), indicating an increase in overall PDE activity. We found that the increase in PDE activity by DETA NONOate was mostly due to PDE3, with a significant decrease in 5′AMP released with the addition of milrinone (Figure 1c). Consistent with the increased expression of PDE3A in DETA NONOate‐treated hPASMC, PDE3‐specific activity in hPASMC was significantly increased in response to DETA NONOate treatment (Figure 1d). An approximate threefold increase in PDE3 activity relative to the vehicle control was observed at 24 hr (*p = *.002) and 48 hr (*p < *.001) following DETA NONOate treatment (Figure 1d).

cAMP is hydrolyzed to AMP by PDE3. Therefore, to verify PDE3 activity, a competitive ELISA was performed in which free cAMP in hPASMC lysates was measured. Cells were treated with DETA NONOate, milrinone, DETA NONOate + milrinone, or vehicle control. cAMP concentrations were significantly reduced in DETA NONOate‐treated cells when compared to vehicle‐treated hPASMC (*p* < .002, Figure 1e). Treatment of cells with the PDE3 inhibitor milrinone resulted in significantly greater cAMP concentrations than compared to both vehicle‐treated hPASMC (*p* < .001) and DETA NONOate‐treated cells (*p* < .001; Figure 1e). The addition of milrinone to DETA NONOate‐treated cells resulted in significantly greater cAMP concentrations than in DETA NONOate‐treated cells (*p < *.001), but the cAMP levels were lower than in those cells treated with milrinone alone (*p < *.03, Figure 1e).

### 3.2 DETA NONOate increased the expression and activity of AMPK

**Figure 2:**
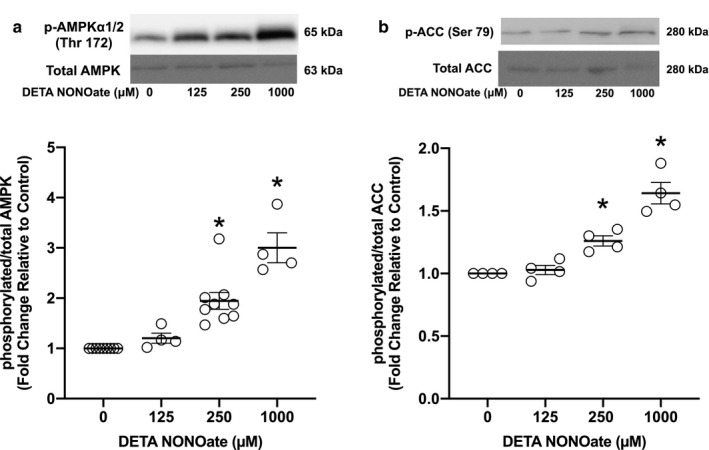
NO activated AMPK. Human PASMC were treated with increasing concentrations of the NO donor, DETA NONOate, for 48 hr. Protein was harvested, analyzed by Western blot, and expression quantified by densitometry. Representative Western blots with data shown below as fold changes ± *SEM* relative to vehicle control for (a) p‐AMPK and total AMPK; *n* = 4–11 for each group, *p < .001 different from control (one‐way ANOVA) and (b) p‐ACC and total ACC; *n* = 4 for each group, *p < .016 different from control (one‐way ANOVA)

AMPK is implicated in modulating pulmonary vascular proliferation and tone; however, the role of NO on AMPK activation in hPASMC is currently unknown. To address this issue, hPASMC were treated for 48 hr with the NO donor, DETA NONOate, to define the effect of NO on AMPK activation. Interestingly, the addition of the NO donor increased the p/T‐AMPK ratio, an index of AMPK activity, in a dose‐dependent manner. An approximate two‐ and threefold greater p/T‐AMPK protein levels were observed following the addition of 250‐μM and 1‐mM DETA NONOate, respectively (*p* < .001, Figure 2a). In support of these findings, p/T‐ACC, a downstream target of AMPK often used as a surrogate of AMPK activity, was significantly increased with DETA NONOate in a dose‐dependent manner, similar to AMPK activity (*p* < .016, Figure 2b).

### 3.3 Pharmacologic inhibition of PDE3 prevented AMPK activation

**Figure 3:**
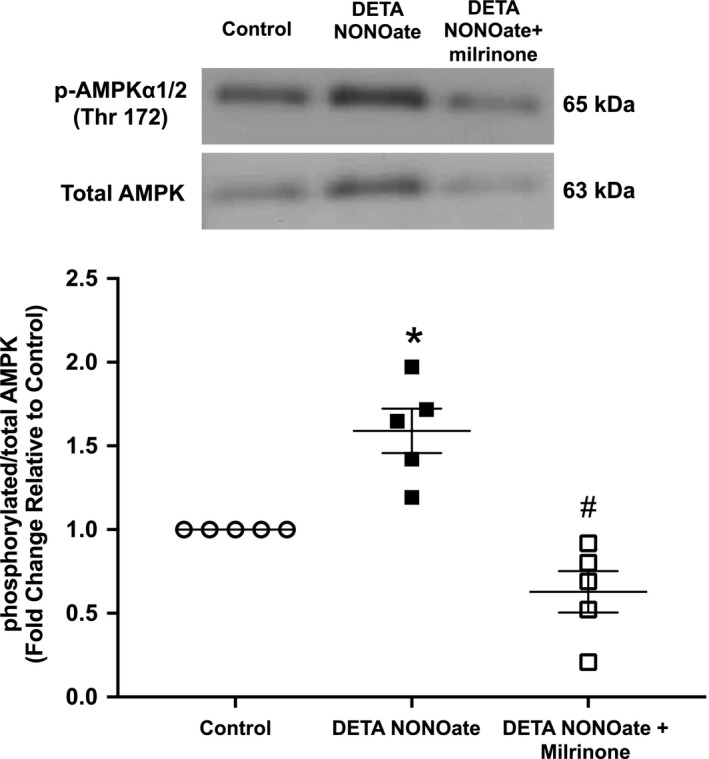
Milrinone blunted NO‐induced AMPK activation. Human PASMC were pretreated for 30 min with milrinone (10 μM), then incubated with DETA NONOate (250 μM) for 48 hr. Cells were treated with milrinone (10 μM) a second time at the 24‐hr time point. Protein was harvested, analyzed by Western blot, and expression quantified by densitometry. Representative Western blots with data shown below as fold changes ± *SEM* relative to vehicle control; *n* = 5 for each group, *p < .005 different from control, #*p* < .001 different from DETA NONOate (one‐way ANOVA)

PDE3 has been shown to regulate AMPK activation in other cell types (Omar et al., 2009; Suzuki et al., 2008). Thus far, our data suggest that AMPK activation may be regulated by PDE3 in hPASMC. To delineate the relationships among NO, PDE3, and AMPK activation, hPASMC were treated with DETA NONOate ± milrinone (PDE3 inhibitor), and p‐AMPK and T‐AMPK protein levels were assessed by Western blot and quantified by densitometry. As expected, the addition of DETA NONOate to hPASMC resulted in a significant increase in p/T‐AMPK protein ratios, when compared to control cells (*p* < .005, Figure 3) suggesting enhanced AMPK activation. In contrast, pretreatment with milrinone prevented the NO‐induced AMPK activation (*p* < .001, Figure 3).

### 3.4 Silencing PDE3A blunted NO‐induced AMPK activation

**Figure 4:**
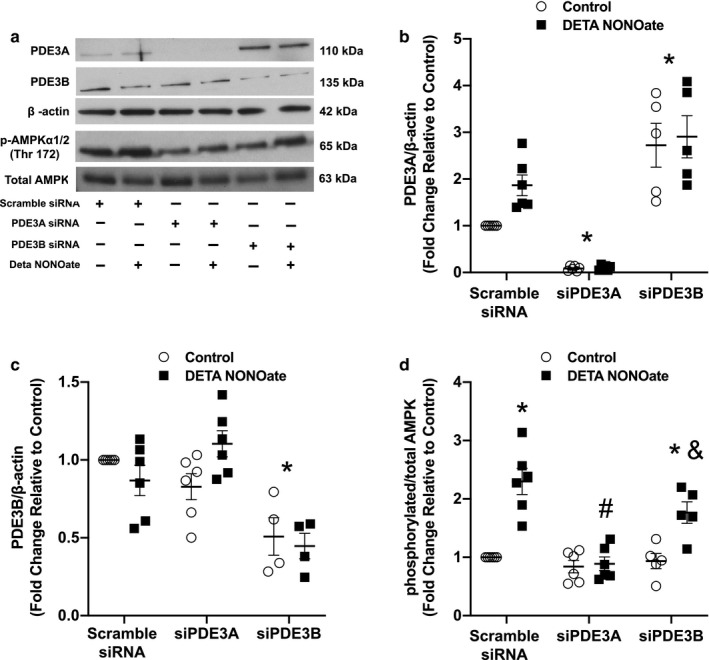
siRNA silencing of PDE3A (siPDE3A) blunted NO‐induced AMPK activation. Human PASMC were transfected with siPDE3A, siPDE3B, or scramble siRNA. After 48 hr, cells were treated with DETA NONOate (250 μM) for 24 hr. Protein was harvested, analyzed by Western blot, and expression quantified by densitometry. (a) Representative Western blots for each PDE3A, PDE3B, β‐actin, and p‐ and total AMPK. Data are shown as fold changes ± *SEM* relative to scramble siRNA control for (b) PDE3A/β‐actin after siPDE3A or siPDE3B transfection; *n* = 5–6 for each group, *p < .025 different from scramble siRNA (two‐way ANOVA). (c) PDE3B/β‐actin after siPDE3A or siPDE3B transfection; *n* = 6 for each group, *p < .005 different from scramble siRNA (two‐way ANOVA), and (d) p/T‐AMPK after siPDE3A or siPDE3B transfection; *n* = 5–6 for each group, *p < .02 different from scramble siRNA, control; #*p* < .001 different from scramble siRNA, DETA NONOate; &*p* < .003 different from siPDE3A, DETA NONOate (two‐way ANOVA)

We induced gene silencing by siRNA transfection to investigate the contributions of PDE3A and PDE3B in AMPK regulation in hPASMC. PDE3A, PDE3B, p‐AMPK, T‐AMPK, and β‐actin were examined by Western blot and quantified by densitometry, following DETA NONOate treatment of hPASMC transfected with siPDE3A, siPDE3B, or scramble siRNA. As expected, PDE3A protein was almost completely undetectable in cells transfected with siPDE3A, while PDE3A knockdown had no substantial effect on PDE3B protein levels (*p* = .24, Figure 4a,c). Surprisingly, there were significantly increased PDE3A protein levels (*p* < .015) following transfection of siPDE3B, despite an incomplete, although statistically significant, knockdown of PDE3B protein levels (Figure 4a,b). Furthermore, transfection of PDE3A siRNA, but not PDE3B siRNA, blunted the NO‐induced AMPK activation (*p* < .001, Figure 4a,d). Taken together, these results indicate that PDE3A plays an important role in AMPK activation by NO in hPASMC.

### 3.5 Regulation of PDE3A expression and AMPK activation by nitric oxide was dependent on the sGC pathway

**Figure 5:**
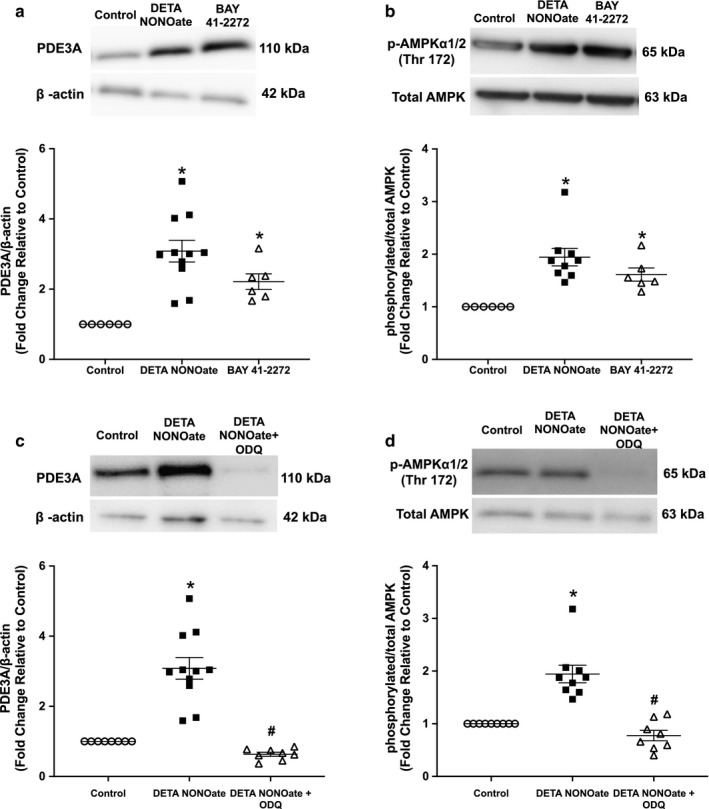
sGC stimulator increased PDE3A protein expression and AMPK phosphorylation, whereas sGC inhibitor prevented the NO‐induced increase in PDE3A protein expression and AMPK phosphorylation in hPASMC. Human PASMC were treated with the NO donor DETA NONOate (250 μM) or sGC stimulator, BAY 41‐2272 (10 μM). Protein was harvested, analyzed by Western blot, and expression quantified by densitometry. Representative Western blots with data shown below as fold changes ± *SEM* relative to vehicle control for (a) PDE3A and β‐actin; *n* = 6–11 for each group, *p < .04 different from control (one‐way ANOVA) and (b) p‐AMPK and total AMPK; *n* = 6–9 for each group, *p < .03 different from control (one‐way ANOVA)l. Alternatively, hPASMC were pretreated for 4 hr with the sGC inhibitor, ODQ (10 μM), then incubated with DETA NONOate (250 μM) for 48 hr. Representative Western blots with data below shown as fold changes ± *SEM* relative to vehicle control for (c) PDE3A and β‐actin; *n* = 8–11 for each group, *p < .001 different from control, #*p* < .001 different from DETA NONOate (one‐way ANOVA) and (d) p‐AMPK and total AMPK; *n* = 8–9 for each group, *p < .001 different from control, #*p* < .001 different from DETA NONOate (one‐way ANOVA)

cGMP acts as a competitive inhibitor of cAMP hydrolysis by PDE3. NO activates sGC, which converts GTP to cGMP. Therefore, we examined whether NO‐induced PDE3A protein expression is mediated through sGC in hPASMC. To this end, hPASMC were treated with BAY 41‐2272 (sGC agonist) or vehicle control for 48 hr, or pretreated with ODQ (sGC inhibitor) for 4 hr prior to the addition of DETA NONOate, followed by a 48‐hr incubation. PDE3A expression and AMPK protein activation were examined by Western blot analysis and quantified by densitometry. Similar to DETA NONOate, treatment with BAY 41‐2272 resulted in greater PDE3A (*p* < .04) and p/T‐AMPK protein expression (*p* < .03, Figure 5a,b). Conversely, treatment with ODQ attenuated the NO‐induced increase in PDE3A protein levels (*p* < .001), and a similar blunting effect was observed for p/T‐AMPK protein expression (*p* < .001 Figure 5c,d).

### 3.6 Treatment with NO donor, AMPK agonist, or PDE3 inhibitor decreased hPASMC proliferation

**Figure 6:**
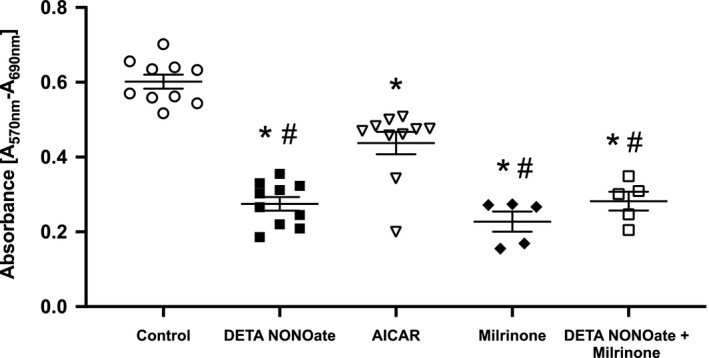
Treatment with NO, AMPK agonist, and PDE3 inhibitor decreased hPASMC proliferation. Human PASMC were treated with DETA NONOate (250 μM), the AMPK agonist, AICAR (5 μM), the PDE3 inhibitor, milrinone (10 μM), the combination of DETA NONOate + milrinone, or vehicle and incubated for 48 hr. MTT proliferation assay was performed. Data shown are means ± *SEM*. *n* = 6–10, *p < .001 different from control, #*p* < .003 different from AICAR (one‐way ANOVA)

Given the conflicting data regarding the role of AMPK in vascular proliferation, the effects of the AMPK agonist, AICAR, as well as NO donor and PDE3 inhibitor on numbers of viable hPASMC were determined in our cell culture model. Viable cell numbers were measured using an MTT assay. Our results indicate that treatment with DETA NONOate, AICAR, or milrinone resulted in lower MTT values consistent with fewer viable cells and decreased proliferation of hPASMC compared to vehicle‐treated controls (*p* < .001, Figure 6). Both DETA NONOate and milrinone treatments decreased hPASMC proliferation more significantly than with AMPK activation (*p* < .001, Figure 6). Combination treatment with DETA NONOate and milrinone did not have any further effect on hPASMC proliferation than either agent alone (Figure 6).

## 4 DISCUSSION

To further understand the mechanisms that underlie therapies used in PPHN, we investigated the role of NO on PDE3 activity in hPASMC. We demonstrate that in hPASMC (1) NO increased PDE3A protein expression and PDE3 activity; (2) NO decreased cAMP concentrations; (3) NO increased p/T‐AMPK and p/T‐ACC protein expression; (4) pharmacologic PDE3 inhibition and siRNA knockdown of PDE3A attenuated NO‐induced AMPK activation; (5) similar to the NO donor, the sGC stimulator increased the PDE3A protein expression and AMPK activation, while inhibition of sGC in NO‐treated cells blunted this effect; and (6) treatment with an NO donor, an AMPK agonist, or a PDE3 inhibitor decreased hPASMC proliferation. Taken together these findings support our hypothesis that in hPASMC, NO increases PDE3 activity by enhancing PDE3A protein expression, which in turn activates AMPK via a sGC‐cGMP‐mediated pathway.

PDE3A and PDE3B are products of distinct genes on different chromosomes and are expressed in diverse tissues (Fagerberg et al., 2014; Manganiello et al., 1995). PDE3A is the predominant isoform found in the cardiovascular system, vascular smooth muscle, and platelets (Fagerberg et al., 2014; Manganiello et al., 1995). As such, PDE3A is implicated in cardiac contractility and systemic vascular smooth muscle cell proliferation (Manganiello et al., 1995; Murray et al., 2002). Conversely, PDE3B is expressed in adipose tissue, pancreatic beta cells, and the liver, and has been implicated in mitochondrial function, as well as in the regulation of energy homeostasis (Choi et al., 2006; Chung et al., 2017; Fagerberg et al., 2014). Our present study found that exposure of hPASMC to an NO donor increased PDE3A protein expression and PDE3 activity with a corresponding decrease in cAMP concentrations at 48 hr (Figure 1). These findings are consistent with similar observations in other studies. For example, in a study of rat PASMC, the authors observed increased PDE3 activity and PDE3A mRNA and protein expression in the NO treatment group (Busch et al., 2010). Similarly, in an ovine model, pulmonary artery PDE3 activity was highest in 1‐day‐old lambs ventilated with 100% O_2_ and NO, and PDE3 activity levels were directly associated with the relaxation response to milrinone (Chen, Lakshminrusimha, et al., 2009). Based on these data, we speculate that the NO‐induced increase in PDE3 activity as a result of greater PDE3A protein expression may serve as a feedback control mechanism to balance cyclic nucleotide concentrations. Given that cGMP competes with cAMP for hydrolysis by PDE3, it may be that the increase in PDE3 activity via the activation of the NO‐cGMP pathway is compensating for the higher levels of cGMP in order to maintain a physiologic balance and preserve baseline vascular tone. Indeed, we found lower cAMP levels in NO‐treated hPASMC (Figure 1e). Our findings support the notion that further studies examining PDE3A expression and activity are warranted to understand the mechanistic role of PDE3A expression and activity in regulating the response to inhaled NO in PPHN.

AMPK is a serine‐threonine kinase that acts as a critical sensor of cellular energy homeostasis in many tissues and has recently been implicated in pulmonary hypertension (Chung et al., 2017; Fisslthaler & Fleming, 2009; Hardie, 2011a; Lai et al., 2016; Teng et al., 2013). There are several known mechanisms by which AMPK is activated; however, the isoform‐specific PDE3 regulation of AMPK in the pulmonary vasculature remains unclear. In other cell types, it has been reported that PDE3B plays a role in AMPK activation by regulating intracellular cAMP concentrations (Chung et al., 2017; Hardie, 2011b; Omar et al., 2009). Specifically in adipocytes, stimulation of cAMP production by the β‐adrenergic agonist isoproterenol increases AMPK phosphorylation, while insulin‐mediated activation of PDE3B prevents the isoproterenol‐induced AMPK phosphorylation (Omar et al., 2009). Pharmacologic inhibition of PDE3 resulted in a reversal effect of insulin on isoproterenol‐induced AMPK phosphorylation (Omar et al., 2009). Interestingly, our data do not support a cAMP‐mediated activation of AMPK by PDE3. We found that although NO increased AMPK phosphorylation (Figure 2a), decreased cAMP levels were observed, presumably due to the increase in PDE3 activity (Figure 1e). In addition, milrinone prevented the NO‐induced AMPK phosphorylation (Figure 3), despite higher cAMP levels compared to NO‐treated hPASMC (Figure 1e). These data suggest that perhaps the NO‐induced AMPK phosphorylation is a result of elevated AMP levels due to greater cAMP degradation from increased PDE3 activity. An increase in the ratio of intracellular AMP to ATP has been shown to enhance phosphorylation and activation of AMPK in response to metabolic stressors, such as hypoxia, resulting in deactivation of energy‐consuming processes and activation of energy‐producing processes, including fatty acid oxidation (Hardie, 2011a). Other mechanisms of AMPK regulation include activation by liver kinase B1 (LKB1), a tumor suppressor (Woods et al., 2003), and activation by Ca^2+^/calmodulin‐dependent protein kinase kinase (CaMKK) as a response to elevated cellular Ca^2+^ (Woods et al., 2005). Further studies will need to be performed to elucidate the exact mechanism of NO‐induced AMPK activation in hPASMC.

To the best of our knowledge, our results are the first to demonstrate the isoform‐specific regulation of AMPK by PDE3A in hPASMC. In adipocytes, overexpression of PDE3B has been shown to inhibit isoproterenol‐induced AMPK phosphorylation (Omar et al., 2009). Contrary to these findings, we demonstrated that siRNA knockdown of PDE3A resulted in the attenuation of NO‐induced AMPK phosphorylation (Figure 4d). These data suggest that PDE3A plays a more critical role in the regulation of NO‐induced AMPK activation than PDE3B in hPASMC; however, there is likely a coordinated effect of both isoforms. Although knockdown of PDE3B by siRNA transfection in hPASMC did not prevent NO‐induced AMPK phosphorylation (Figure 4a,d), there was still the presence of a low level of PDE3B protein expression that might be functionally active. Interestingly, despite the incomplete knockdown of PDE3B, PDE3A protein levels were significantly greater in the siPDE3B‐transfected group compared to scramble controls (Figure 4a,b). This increase in PDE3A may be a compensatory response to maintain PDE3 activity in the face of decreased PDE3B protein levels, and the reason NO‐induced AMPK activation was not prevented by PDE3B knockdown. In other words, it is possible that PDE3B also plays a role in AMPK regulation that was not evident in our study, either directly through the actions of the PDE3B protein or indirectly by increasing PDE3A protein levels, as we observed after PDE3B siRNA transfection.

Furthermore, we aimed to delineate the mechanism by which NO increases PDE3A protein expression and specifically examine whether the increase in PDE3A occurs via the NO‐sGC‐cGMP pathway. Therefore, we treated hPASMC with the sGC agonist, BAY 41‐2272, or a selective sGC inhibitor, ODQ, in combination with NO. Stimulation of sGC with BAY 41‐2272 increased the PDE3A protein expression, whereas inhibition of sGC with ODQ prevented the NO‐induced increase in PDE3A expression (Figure 5a,c). These findings are consistent with previous reports demonstrating a 50% reduction in aortic PDE3A protein expression in GC knockout mice (global and smooth muscle cell‐specific; Dünnes et al., 2018). Similarly, rat PASMC treated with various sGC stimulators showed an increase in PDE3A gene expression (Busch et al., 2010). These data infer that NO increases PDE3A protein levels via a mechanism dependent upon the NO‐sGC‐cGMP pathway. The exact mechanism through which the NO‐sGC‐cGMP pathway upregulates PDE3A protein levels remains to be elucidated in future studies. Since NO increases cGMP levels, it is possible that the increase in PDE3 activity by NO represents a feedback control mechanism to maintain homeostasis in the concentration and availability of the cyclic nucleotides, cAMP and cGMP. Given the importance of PDE3 in the regulation of the two critical vasodilatory second messengers cAMP and cGMP, our findings raise the possibility that PDE3A dysregulation could be implicated in the pathogenesis of PPHN.

Finally, we investigated the role of NO, AMPK activation, and PDE3 inhibition on hPASMC proliferation. We found that NO demonstrated potent antiproliferative effects on hPASMC (Figure 6), which have been well established in the literature (Mizuno et al., 2004). Cornwell, Arnold, Boerth, and Lincoln (1994) demonstrated that the NO donor, SNAP, produced large increases in cGMP and inhibited DNA synthesis and cell proliferation in rat aortic smooth muscle cells by increasing cAMP‐dependent protein kinase activity without changes in cAMP levels. We found lower cAMP levels in hPASMC following treatment with DETA NONOate. These findings suggest that the antiproliferative effect of NO is complex and not dependent on cAMP levels. Whereas NO acts as both a target and an effector of the AMPK pathway in endothelial cells (Fisslthaler & Fleming, 2009; Suzuki et al., 2008), less is known about the mechanism by which NO regulates AMPK in smooth muscle cells. Moreover, data on the role of AMPK in vascular proliferation and pulmonary hypertension are conflicting (Agard et al., 2009; Dean, Nilsen, Loughlin, Salt, & MacLean, 2016; Ibe et al., 2013; Moral‐Sanz et al., 2016; Salt & Hardie, 2017; Song et al., 2016). Several studies have shown that AMPK promotes SMC proliferation and the development of pulmonary hypertension, which was reversed with AMPK inhibition (Ibe et al., 2013; Moral‐Sanz et al., 2016). Conversely, other studies have shown that AMPK activation reduced SMC proliferation and vascular remodeling and may be protective against the development of hypoxia‐induced pulmonary hypertension (Agard et al., 2009; Dean et al., 2016; Song et al., 2016). Importantly, in our present study, hPASMC proliferation was decreased with AMPK activation by AICAR (Figure 6). Although our data demonstrate that PDE3 inhibition prevented NO‐induced AMPK activation, pretreatment with the nonselective PDE3 inhibitor milrinone also decreased hPASMC proliferation, similar to milrinone treatment alone (Figure 6). Previous in vitro studies have shown that decreasing cAMP degradation (PDE inhibitors) or increasing cAMP synthesis (adenylyl cyclase activator) significantly inhibits vascular smooth muscle cell proliferation (Chen, Calvert, Meng, & Nelin, 2012; Smith, Newby, & Bond, 2019). Thus, we speculate that the decreased proliferation observed with milrinone treatment is likely due to the increase in cAMP levels (Figure 1e) and is consistent with previous reports demonstrating that nonselective PDE3 inhibition decreases systemic and pulmonary vascular SMC proliferation (Chen et al., 2012; Kondo, Umemura, Miyaji, & Nakashima, 1999; Phillips, Long, Wilkins, & Morrell, 2005). It is evident that the antiproliferative effects of PDE3 inhibition and the associated increase in cAMP levels are independent of AMPK activation. Thus, we speculate that PDE3 inhibition and AMPK activation reduce hPASMC proliferation through different biochemical pathways. For example, cAMP has been shown to decrease cellular proliferation via inhibition of the mitogen‐activated protein kinase cascade (Schmitt & Stork, 2001), whereas AMPK activation has been shown to decrease proliferation via upregulation of p53 protein and suppression of mammalian target of rapamycin signaling (Motoshima, Goldstein, Igata, & Araki, 2006). Our MTT studies support that hPASMC proliferation is complex and likely involves the contribution of multiple signaling pathways. Determining the underlying mechanisms by which the interaction of these agents alter proliferation in hPASMC will require additional studies.

The present study has several limitations. We performed these studies in cultured hPASMC that are often isolated from the proximal vasculature rather than more distal resistance pulmonary arteries. We acknowledge that hPASMC in the resistance pulmonary arteries may have a different phenotype compared to those used and further studies in resistance pulmonary arteries will be necessary. Additionally, it is well recognized that smooth muscle cell communication with endothelial cells via paracrine signaling and direct cellular contact is critical to hPASMC function and to the development of pulmonary hypertension. Therefore, using a co‐culture of endothelial cells and smooth muscle cells may represent a more physiologic method to study the role of PDE3 and the regulation of AMPK in pulmonary hypertension, which are aims for future studies. We studied nonstimulated hPASMC in normoxic conditions, which may not entirely mimic the changes seen in PASMC in pulmonary hypertension. Nevertheless, we studied PDE3 function in nonstimulated cells as a first step in defining the interactions of NO, PDE3, and AMPK and how these interactions contribute to alterations in proliferation. Data obtained will be leveraged to design future studies in which the NO‐PDE3‐AMPK pathway will be examined under conditions associated with pulmonary hypertension, such as hypoxia. Furthermore, as PPHN is generally treated with a combination of hyperoxia and inhaled NO, these interactions will also need to be evaluated in future studies.

In conclusion, our in vitro findings may have important clinical implications. PDE3A and PDE3B serve diverse cell‐specific functions and are distinct in their regulation of AMPK activation. We observed that in hPASMC, PDE3 activity, PDE3A, and p/T‐

AMPK protein expressions were increased following treatment with NO, with a concomitant decrease in cAMP concentrations. Knockdown of PDE3A blunted NO‐induced AMPK activation, indicating an isoform‐specific regulation of AMPK by PDE3. Furthermore, our data suggest that the NO‐induced PDE3 activity and resultant AMPK activation occur via a sGC‐cGMP‐dependent mechanism. The present study increases our understanding of not only the isoform‐specific role of PDE3 in the regulation of NO‐induced AMPK in hPASMC, but also the interactions of two important pathways critical in the maintenance of pulmonary vascular tone. We speculate that increased PDE3A and AMPK by NO have implications in the pathobiology of pulmonary hypertension and may provide rationale for the development of novel isoform‐specific therapeutic targets for the treatment of PPHN.

## CONFLICT OF INTEREST

None of the authors listed on this submission have any conflict of interest, financial, or otherwise to report.

## AUTHOR CONTRIBUTIONS

Dr. Julie Dillard contributed to design of the work, acquisition and interpretation of data for the work, drafting and revising for intellectual content. Xiaomei Meng contributed to design of the work, acquisition of the data, and drafting the work. Dr. Leif Nelin contributed to design of the work, analysis and interpretation of data, and revising for intellectual content. Dr. Yusen Liu contributed to design of the work, analysis and interpretation of data, and revising for intellectual content. Dr. Bernadette Chen contributed to conception of the work, analysis and interpretation of data, and drafting and revising for intellectual content. All authors gave final approval of the version to be published and agree to be accountable for all aspects of the work in ensuring that questions related to accuracy are appropriately investigated and resolved.
